# Supplementary material for: Comparison of multimarker logistic regression models, with application to a genomewide scan of schizophrenia
Source: BMC Genet. 2010 Sep 9;11:80. doi: 10.1186/1471-2156-11-80 (PMC2949738; doi:10.1186/1471-2156-11-80)
Supplement: Additional file 1 — Imputation results (1). Imputation results from first chromosome 9 region. [file 1471-2156-11-80-S1.PDF]

Number of SNPs from region in original dataset	29
Number of SNPs from region in HapMap phase II dataset	140
Number of SNPs with allelic dosage squared correlation ≥ 0.8	41
Number of above SNPs with p-value < 0.05	9
Minimum p-value from above	6.18×10^{-4}
