# Supplementary material for: Comparison of multimarker logistic regression models, with application to a genomewide scan of schizophrenia
Source: BMC Genet. 2010 Sep 9;11:80. doi: 10.1186/1471-2156-11-80 (PMC2949738; doi:10.1186/1471-2156-11-80)
Supplement: Additional file 2 — Imputation results (2). Imputation results from second chromosome 9 region. [file 1471-2156-11-80-S2.PDF]

Number of SNPs from region in original dataset	21
Number of SNPs from region in HapMap phase II dataset	77
Number of SNPs with allelic dosage squared correlation ≥ 0.8	39
Number of above SNPs with p-value < 0.05	9
Minimum p-value from above	7.35×10^{-4}
